# Har Gobind Khorana (1922–2011): Pioneering Spirit

**DOI:** 10.1371/journal.pbio.1001273

**Published:** 2012-02-21

**Authors:** Thomas P. Sakmar

**Affiliations:** Laboratory of Molecular Biology and Biochemistry, The Rockefeller University, New York, New York, United States of America

## Abstract

Thomas Sakmar remembers one of the founding fathers of chemical biology.

One of the great chemists of the 20th century died on November 9, 2011. H. Gobind Khorana, a founder of what we now call chemical biology and a pioneer at the dawn of the age of molecular biology, was 89 (see Image 1).

**Figure:**
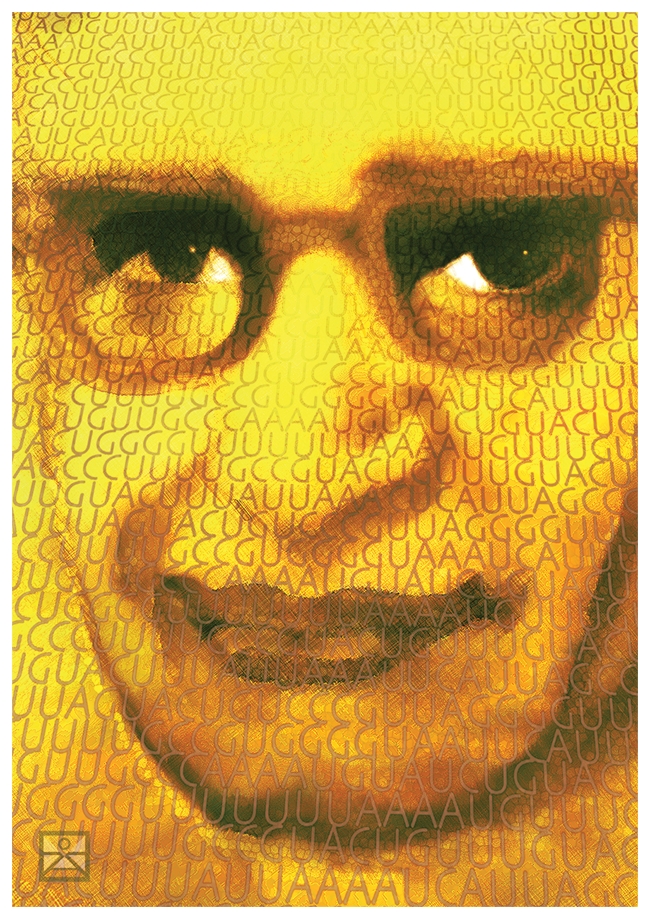
Image 1. Portrait of H. G. Khorana. Image credit: Karina Åberg.

Gobind was a creative and insightful chemist, with many landmark achievements to his credit, including the first practical synthesis of nucleotides and coenzymes in the mid-1950s. He had a pioneering sense of the power of multidisciplinary work and he continued to re-invent himself at regular intervals for more than 50 years.

He shared the Nobel Prize for Physiology or Medicine in 1968 (with Robert W. Holley and Marshall W. Nirenberg), at age 46, for contributions toward elucidating the genetic code—one of the great scientific achievements of the age of molecular biology. Energized by the Nirenberg and Matthaei experiment from 1961, where a cell-free extract produced a protein made entirely of phenylalanine when poly-U was added, Gobind's group at the Institute for Enzyme Research at the University of Wisconsin–Madison worked around the clock in double shifts to synthesize all of the possible triplet tri-nucleotides, thus providing a firm basis to establish the complete codon assignments and to determine how the code was read. Gobind pursued this punishing schedule even though work on the genetic code in the early 1960s represented a digression of sorts from studies aimed at refining procedures to synthesize even longer polynucleotides.

In 1972, Gobind described the total chemical synthesis of a functional tRNA gene in an unprecedented and still unsurpassed achievement in chemical biology, which was published in an entire issue of *Journal of Molecular Biology* in December 1972—15 consecutive articles, 313 consecutive pages. The achievement was even more striking if we stop to consider that when the project was initiated, in 1960, there was no reliable method to synthesize more than a di-nucleotide in reasonable yield, nor was there a way to sequence DNA. The report of “nearest-neighbor” analysis by Arthur Kornberg—a test to confirm the sequence of the bases during replication—was all that Gobind needed to commit to the work [1]. And once Gobind committed, there was no turning back—ever.

By the time I had met Gobind for the first time, at the Massachusetts Institute of Technology (MIT) in 1984, he was 62 years old—already a giant from an earlier time. He gave no impression at that initial meeting that he was satisfied with his achievements or finished with his work. And in fact, he wasn't. He would be active for 23 years more and would publish seminal work on transmembrane signaling and energy transduction.

I remember vividly that first meeting, and I guess that anyone who has ever met Gobind would remember him. He was not necessarily what you would expect given his achievements. He seemed to have time; he listened and had no air of pretense at all. He was a professor who did not profess; an expert who did not claim to know all there was to know. He preferred to be on the receiving end, to acquire more information to store, synthesize, and later recall using his prodigious memory.

Of course, I already knew about his work on the genetic code and gene synthesis when we met. And I had studied the curious photo of him from 1966 at the Cold Spring Harbor Symposium on the Genetic Code that was reprinted in *Recombinant DNA: A Short Course* [2]. Wearing a grey suit and dark tie, Gobind is facing the camera and is caught just as he had apparently reached up to sweep back his hair (see Image 2). Standing about two paces to the right are Francis Crick and Marianne Grunberg-Manago, but they are looking past Gobind into the distance. Gobind, at least in earlier years, must have been easy to underestimate and overlook. And though an unassuming and humble man, at times shy, he was definitely not reserved or impersonal.

**Figure:**
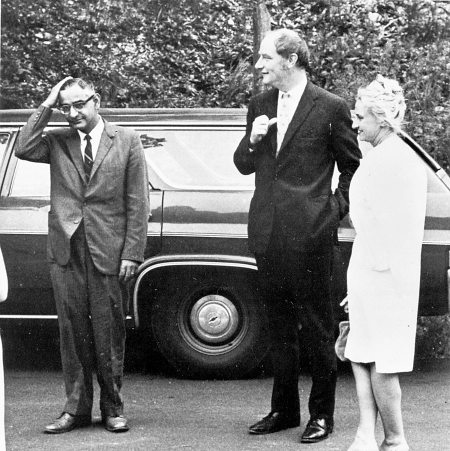
Image 2. H. G. Khorana. Photo from Cold Spring Harbor Laboratory.

With an intellectual audacity that propelled him forward, Gobind would study and seek out the leaders of each successive field of interest and then, in most cases, leap beyond them while leaving a trail of highly accomplished colleagues (perhaps 250 postdoctoral fellows and students over the years), who would often go on to become leaders in whatever field he had jettisoned earlier.

He thought constantly about effort and commitment, sometimes doubling down when projects seemed hopeless. But he also seemed to know when to quit, or better stated, when to move on to other work, sometimes related, but often totally new.

The best example of this is when he switched from working primarily on nucleic acids and gene synthesis and took up the challenging subject of membrane proteins in the mid-1970s. After nearly a year traveling and visiting the leaders in the field, he chose to work on bacteriorhodopsin, the light-driven proton pump from patches known as the purple membrane. Within about 5 years, he would publish its complete amino acid sequence, the first integral membrane protein to be sequenced. He then cloned the gene, worked out a heterologous expression scheme, and used site-directed mutagenesis (just invented by his former colleague Michael Smith) to elucidate mechanism. Related work on the G protein–coupled receptor (GPCR), rhodopsin, would follow. And many of the methods he reported, including the use of immunoaffinity purification and other approaches, were used later to advance the structural biology of GPCRs.

Only later did I learn the personal and professional life story of this remarkable man—his almost unfathomable journey from an impoverished village in Punjab, where his father taught him to read and helped to establish a single-room school. How as a child he woke up early to go out into the village and look for a house with smoke coming out of the chimney, and then ask for a bit of ember to take home to light the cooking fire. How later he would sit on the steps of the post office and transcribe letters for illiterate townspeople. It was there that he undoubtedly developed his characteristic micro-script handwriting, which anyone who ever worked with him on a manuscript draft would instantly recognize.

There are many apocryphal stories about Gobind's early formation in science. But the short time he spent at Eidgenössische Technische Hochshule in Zurich in 1948 with Vladimir Prelog was the most important, and probably most difficult, period in his life. He came to Zurich without letter of introduction or reference and just walked in on Prelog. Based on a quick review of his dissertation on alkaloid synthesis and melanin from the laboratory of Roger J. S. Beer at Liverpool, which Gobind eagerly presented, Prelog accepted him as a postdoctoral fellow, but no funding was forthcoming. Gobind essentially lived in the laboratory on rice and unpasteurized milk for the next 11 months (official reports say that he subsisted on “savings.”) Despite the hardships, Gobind formed an instant and long-lasting bond with Prelog, a legendary mentor whom Gobind credits for influencing his approach to work and his integrity as a scientist.

Ironically, it was Gobind's initial lack of progress at the bench that serendipitously led to his eventual spectacular success. He spent hours in the library reviewing the German organic chemistry literature, and came across a description of a little-known synthetic reagent, carbodiimide, that had been essentially forgotten and had never surfaced in the English literature. Though of no use to his work at the time, Gobind would remember the reagent years later and apply it to create a revolution in biochemistry.

In 1949 Gobind was required to return to India to fulfill the requirements of service mandated by his earlier scholarship, but in post-partition India his ancestral village ended up in Pakistan and his family had dispersed. Unable to find work and living in the servants' quarters of his uncle's house in New Delhi, Gobind became essentially an academic refugee, spending a fruitless year looking for work. Thankfully, the government annulled the bond to repay his scholarship and he accepted a fellowship to work with Alexander R. (Lord) Todd at Cambridge, England, thanks to the help of Cambridge professor G. W. Kenner, whom Gobind had met in Zurich.

So at age 27, Gobind returned to England after his extended family scraped together the fare for ship's passage. Although Kenner and Khorana initially used carbodiimide reagents to activate the carboxyl-terminal ends of peptides, the zeitgeist at Cambridge favored nucleic acids, and Todd had just deciphered the correct chemical linkages between nucleotides in DNA.

As luck would have it, Gordon M. Shrum, head of the British Columbia Research Council, visited Cambridge and asked Todd to suggest a chemist who might be willing to move to Vancouver to start a new non-academic research laboratory—no start-up package, but unlimited freedom. Todd suggested Gobind, who, in 1952, married Esther Silber, a Swiss woman he had met in Prague in 1947, and moved with her to Vancouver. Gobind and Esther raised three children there and Esther provided a foundation and bearing for Gobind that sustained him for the next nearly 50 years.

Gobind spent much of that first year in Vancouver writing a major scholarly review on protein structure determination, then remarkably set it aside to work on nucleic acids. The real turning point came in 1954 when Gobind published the synthesis of ADP and ATP using the carbodiimide reaction [3]. He later synthesized cyclic-nucleotides, asymmetric di-nucleotides, and other molecules of biological significance. During summer “vacations” notable scientists including Paul Berg, A. Kornberg, Eugene Kennedy, and many others, visited Gobind's laboratory to learn how to prepare and use the new carbodiimide reagents.

Over time with the help of several notable colleagues (Gordon W. Tener, John G. Moffat, M. Smith, and others), every significant known nucleotide and nucleotide co-factor was synthesized, with the culmination in 1960 of the synthesis of coenzyme A, by far the most complex of the nucleotide cofactors. In one of the most remarkable concluding statements in the chemical literature, Moffat and Khorana wrote [4]:

The synthesis of Coenzyme A and the work reported in the accompanying papers bring to a close the program of research initiated in this Laboratory some seven years ago on the development of methods for the synthesis of unsymmetrical pyrophosphates of biological interest. The methods available are believed to be satisfactory and completely general …

Why would someone at the top of his game declare that the program was over? Gobind had something else in mind. He moved to the Enzyme Institute at the University of Wisconsin–Madison, his sights set even higher: chemical synthesis of a gene.

His line of thinking was clearly outlined in 1960, and if I had to assign a clear date to the origins of chemical biology as a new discipline, this would be it [5]:

In the distant future, the total chemical synthesis of macromolecules possessing biological function must also be considered. The problems are vastly more complex than anything previously undertaken by experimental organic chemistry. Nevertheless, Biology urgently asks: Will Organic Chemistry extend its horizons and accept the challenge?

As a member of Gobind's group at MIT in the late 1980s, I saw a man still totally focused on laboratory work, following an almost relentless schedule, three or four group meetings a week, plus Saturday tea. Most of the meetings were shared jointly with the laboratory of Uttam L. RajBhandary, who, as a long-term colleague and constant presence at MIT, contributed immeasurably to Gobind's success and longevity after 1970.

I would later realize that my colleagues at MIT were carefully chosen for their drive and perseverance, as much as for their technical expertise. Almost everyone had some connection to someone Gobind knew personally, but of course, Gobind knew just about everyone.

Gobind exuded an almost childlike enthusiasm and energy, but with a laser-like focus. He would listen intently, tilting his head ever so slightly and leaning in toward you. Then something would click and he would leap up from his chair and bound toward a filing cabinet. Within seconds he would pull out a relevant set of notes, or a reprint. His files were almost exclusively organized by scientists' names—no need for key words or alphabetical subject lists. For Gobind, science was intensely personal, but it was not only about the great scientists whom had influenced him directly, most notably V. Prelog, A. R. Todd, F. Crick, A. Kornberg, P. Berg, George Beadle, Fritz Lipmann, and many others. It was also about scientists who were doing careful work mainly at the boundaries between fields, scientists who might help him achieve his goals.

My association and friendship with Gobind formed the foundation of my scientific career. With Gobind's death, I feel an immense sense of loss, and I grieve the passing of a truly great man.

At the end of the epilogue of his book entitled *Chemical Biology* [6], H. Gobind Khorana, the great chemist and innovator, quoted these lines that I can imagine he read as a young man before leaving his home village on an incredible personal and scientific journey:

We must be still and still moving

Into another intensity

For a further union, a deeper communion

Through the dark cold and the empty desolation,

The wave cry, the wind cry, the vast waters

Of the petrel and the porpoise. In my end is my beginning.

—T. S. Eliot
